# Findings from a pilot Randomised trial of an Asthma Internet Self-management Intervention (RAISIN)

**DOI:** 10.1136/bmjopen-2015-009254

**Published:** 2016-05-12

**Authors:** D Morrison, S Wyke, K Saunderson, A McConnachie, K Agur, R Chaudhuri, M Thomas, N C Thomson, L Yardley, F S Mair

**Affiliations:** 1 General Practice & Primary Care, Institute of Health & Wellbeing, University of Glasgow, Glasgow, UK; 2 Institute of Health and Wellbeing/Interdisciplinary Research Professor, College of Social Sciences, University of Glasgow, Glasgow, UK; 3 Robertson Centre for Biostatistics, Institute of Health and Wellbeing, University of Glasgow, Glasgow, UK; 4 Institute of Infection, Immunity and Inflammation, Gartnavel General Hospital, University of Glasgow, Glasgow, UK; 5 Primary Care Research, Aldermoor Health Centre, University of Southampton, Southampton, UK; 6 Department of Psychology, University of Southampton, Southampton, UK

**Keywords:** Internet, PRIMARY CARE, Adherence, behaviour change, complex intervention

## Abstract

**Objective:**

To evaluate the feasibility of a phase 3 randomised controlled trial (RCT) of a website (Living Well with Asthma) to support self-management.

**Design and setting:**

Phase 2, parallel group, RCT, participants recruited from 20 general practices across Glasgow, UK. Randomisation through automated voice response, after baseline data collection, to website access for minimum 12 weeks or usual care.

**Participants:**

Adults (age≥16 years) with physician diagnosed, symptomatic asthma (Asthma Control Questionnaire (ACQ) score ≥1). People with unstable asthma or other lung disease were excluded.

**Intervention:**

‘Living Well with Asthma’ is a desktop/laptop compatible interactive website designed with input from asthma/ behaviour change specialists, and adults with asthma. It aims to support optimal medication management, promote use of action plans, encourage attendance at asthma reviews and increase physical activity.

**Outcome measures:**

Primary outcomes were recruitment/retention, website use, ACQ and mini-Asthma Quality of Life Questionnaire (AQLQ). Secondary outcomes included patient activation, prescribing, adherence, spirometry, lung inflammation and health service contacts after 12 weeks. Blinding postrandomisation was not possible.

**Results:**

Recruitment target met. 51 participants randomised (25 intervention group). Age range 16–78 years; 75% female; 28% from most deprived quintile. 45/51 (88%; 20 intervention group) followed up. 19 (76% of the intervention group) used the website, for a mean of 18 min (range 0–49). 17 went beyond the 2 ‘core’ modules. Median number of logins was 1 (IQR 1–2, range 0–7). No significant difference in the prespecified primary efficacy measures of ACQ scores (−0.36; 95% CI −0.96 to 0.23; p=0.225), and mini-AQLQ scores (0.38; −0.13 to 0.89; p=0.136). No adverse events.

**Conclusions:**

Recruitment and retention confirmed feasibility; trends to improved outcomes suggest use of Living Well with Asthma may improve self-management in adults with asthma and merits further development followed by investigation in a phase 3 trial.

**Trial registration number:**

ISRCTN78556552; Results.

**Strengths and limitations of this study**

- A recent UK review of asthma deaths showed many could have been avoided if medication management and other self-management strategies had been better, so finding optimum approaches to support self-management of asthma is critical and digital interventions show promise.
- The ‘Living Well with Asthma’ website was iteratively designed with input from experts in asthma, self-management support, behaviour change and adults with asthma themselves; it aims to support optimal medication management, promote use of action plans, encourage attendance at asthma reviews and increase physical activity.
- We conducted a phase 2 parallel group, randomised controlled trial; randomisation was through automated voice response, after baseline data collection but blinding of the researchers or participants at outcome, measurement was not possible.
- Our low response rate is a concern; however, we have described our population in detail (unlike previous reports of digital interventions for asthma self-management), and our baseline characteristics demonstrate that patients were recruited from a range of socioeconomic backgrounds.

## Introduction

Asthma is a common condition affecting over 300 million people worldwide, with increasing global prevalence.^1^ While there are newer pharmacological treatments for individuals with severe asthma,^2–5^ improvements in outcomes for the majority with mild-to-moderate asthma have stalled.^6^ A recent UK review of asthma deaths showed potentially avoidable factors in the majority, particularly relating to self-management and adherence to treatment.^7^

Despite clear evidence that self-management education, asthma action plan use and regular professional review improve outcomes,^8^ translation into everyday practice has proven difficult,^6^ and most patients still lack an action plan and sufficient understanding to self-manage effectively. Poor adherence to regular preventative medication (primarily with inhaled corticosteroids, ICS) is a particular problem. Using digital interventions to promote self-management behaviours shows promise, but uncertainty persists as to the most effective formulation of the intervention and the target population.^9^

In this phase 2, pilot randomised controlled trial (RCT), we evaluated the feasibility and effectiveness of using a low-intensity online intervention aimed at promoting effective self-management (especially adherence to ICS) in adults with mild-to-moderate asthma, compared with usual care. We developed the intervention (‘Living Well with Asthma’) incorporating evidence from the literature and relevant theory. Several phases of user testing in alignment with the ‘person-based approach’ to developing digital behaviour change interventions were undertaken.^10^ Following the Medical Research Council (MRC) guidance on developing and evaluating complex interventions,^11^ our objective was to determine the feasibility of conducting a phase 3 RCT, and obtained initial estimates of effects on outcomes.

## Methods

Our trial protocol is described in detail elsewhere.^12^ A brief summary is provided here.

### Settings and participants

We recruited from 20 general practices in Glasgow, UK, between 23/09/2013 and 21/02/2014, using clinical databases to identify potential participants who were invited by mail to participate and complete the Asthma Control Questionnaire (ACQ). We recruited adults aged 16 years or older, with a physician diagnosis of asthma and ACQ score ≥1, who provided written informed consent. For full inclusion and exclusion criteria see box 1. Our search strategy is shown in the online supplementary data file.

10.1136/bmjopen-2015-009254.supp1 Supplementary data

Box 1 **Inclusion and exclusion criteria**

Inclusion criteria

- Written informed consent
- Age 16 years or older
- Diagnosis of asthma by a health professional, and duration of asthma symptoms >1 year
- Asthma Control Questionnaire score (ACQ; six-questions version) ≥1 suggesting poorly controlled asthma
- Ability to access the internet via desktop or laptop (tablets and smartphones not sufficient)

Exclusion criteria

- Unstable asthma as defined as the presence of one or more of the following events in the 4 weeks prior to randomisation:
  - Asthma-related hospital admission
  - Emergency department attendance for asthma
  - ‘Out of hours’ visit of patients to the general practitioner (GP) for asthma
  - GP visit to patient at home for asthma
- Presence of active lung disease other than asthma
- Mental impairment or language difficulties that make informed consent impossible
- Frequent asthma exacerbations with >4 courses of oral prednisolone in the 12 months prior to randomisation
- Cognitive impairment
- Terminal illness

### Study design overview and intervention description

We conducted a non-blinded pilot RCT of access to the ‘Living Well with Asthma’ website versus usual care for 51 participants. Participants were assessed in their own homes at baseline and at 12 weeks or as soon as possible after this date.

The intervention development is described elsewhere,^13^ but in summary aimed to (1) provide understanding of current level of asthma control and how to improve it, specifically by optimising use of prescribed medication; (2) challenge attitudes and concerns around medications; (3) learn how to get the most out of their annual asthma review; (4) prompt provision and use of a personal asthma action plan from a health professional and (5) send timely reminders for influenza vaccination and reordering refill inhaler prescriptions. The website did not advise medication changes, but suggested contacting a health professional if inadequate control was identified, with clear advice for seeking help in an emergency. The website is interactive, aiming to engage the user in recognising that their asthma is uncontrolled, and illustrate the benefits via case vignettes (based on real life examples) of taking their medications as prescribed. The website is tailored based on their current use of preventer inhalers (never been prescribed; prescribed but do not really use; use regularly). There is a ‘4-week challenge’ that users can sign up to, where they commit to taking their preventer regularly for 4 weeks, are guided through establishing their personal barriers to regular use (see screenshot in online supplementary data file for further illustration) and developing potential solutions to these barriers.

The intervention group was given website login details and a computer link, and advised to use the website as much or as little as they wished (total time to visit all pages once ∼90 min). We developed the website using an open source software package called LifeGuide.^14^ ^15^

### Randomisation and blinding

Randomisation occurred after baseline data collection, using a third party interactive voice response system (IVRS) ensuring allocation concealment. The randomisation schedule was generated in advance of the study by the Robertson Centre for Biostatistics, in a 1:1 ratio, using the method of randomised permuted blocks of length 4, without stratification. Access to the randomisation schedule was restricted to those within the Centre with responsibility for provision of the IVRS. The comparison group was offered access to the intervention after the follow-up visit.

### Primary outcomes

The primary end points were: recruitment and retention rates at follow-up, website use, and changes from baseline for ACQ^16^ and mini-Asthma Quality of Life Questionnaire (AQLQ) scores.^17^ The ACQ and mini-AQLQ have a minimal clinically important difference (MCID) of 0.5.^18^ This pilot study was not powered to detect a difference in these two clinical outcomes; they were included in order to assess feasibility and inform sample size calculations for a future full-scale RCT.

### Secondary outcomes

We evaluated a range of secondary outcomes in order to assess their feasibility for use in a future full-scale RCT.

Individual domains of the mini-AQLQ were reported. These comprise of symptoms, activity limitation, emotional function and environmental stimuli. Knowledge, skills and confidence to manage health was measured via the Patient Activation Measure (PAM).^19^ Self-reported adherence was assessed by both enquiring what proportion of prescribed ICS were actually taken, and via the Morisky Medication Adherence Scale (MMAS).^20^ Airway inflammation is measured by fraction exhaled nitric oxide (FeNO).^21^ Lung function was assessed via prebronchodilator spirometry, including forced expiratory volume in 1 s (FEV_1_); FEV_1_ percentage predicted; and FEV_1_/forced vital capacity. Lung function (spirometry) was performed to the American Thoracic Society (ATS) standards,^21^ ^22^ where possible, and the proportion of tests not meeting these standards recorded. As well as the asthma-specific mini-AQLQ, generic quality of life was measured using the EuroQol (EQ)-5D.^23^ We collected changes to medication use, recorded numbers of health service contacts and severe exacerbations were noted by recording the number of oral prednisolone courses. These data were self-reported. Those in the intervention group received the problematic experience of therapies scale (PETS) to facilitate understandings of barriers to using the website, and following its advice.

### Data analysis

Continuous data are summarised as mean and SD or range, or as median and IQR, and categorical data as counts and percentages. Linear regression was used to estimate differences in continuous outcomes between groups at follow-up, adjusting for baseline scores. Estimated between-group differences are reported with a 95% CI and p value. For continuous outcomes that were not normally distributed, changes from baseline were compared between groups using Wilcoxon rank-sum tests. Categorical variables were compared between groups using Fisher's exact test. All analyses were carried out using SPSS Statistics V.22 and Microsoft Office Excel. The primary analysis was intention-to-treat and involved all patients who were randomly assigned, except with spirometry where only those meeting ATS/European Respiratory Society (ERS) eligibility criteria will be analysed.^22^

## Results

### Baseline characteristics

The groups were largely well matched. Participants were aged between 16 and 78 years, and 75% were female (table 1).

**Table 1:** Baseline demographic characteristics of study population per group

	Overall (n=51)	Comparison (n=26)	Intervention (n=25)
Age (years), mean (SD)	45.5 (15)	46.4 (14)	44.6 (17)
Female, n (%)	38 (75)	20 (77)	18 (72)
Ethnicity			
White, n (%)	48 (94)	24 (92)	24 (96)
Other, n (%)	3 (6)	2 (8)	1 (4)
Smoking status:			
Current, n (%)	5 (10)	2 (8)	3 (12)
Former smoker, n (%)	18 (35)	11 (42)	7 (28)
Never smoked, n (%)	28 (55)	13 (50)	15 (60)
SIMD quintile (1=most deprived, 5=least deprived)			
SIMD 1, n (%)	14 (28)	7 (27)	7 (28)
SIMD 2, n (%)	11 (22)	6 (23)	5 (20)
SIMD 3, n (%)	9 (18)	4 (15)	5 (20)
SIMD 4, n (%)	5 (10)	3 (12)	2 (8)
SIMD 5, n (%)	12 (24)	6 (23)	6 (24)
Employment status			
Employed, n (%)	25 (49)	11 (42)	14 (56)
Unemployed, n (%)	8 (16)	3 (12)	5 (20)
Retired, n (%)	9 (18)	5 (19)	4 (16)
Student, n (%)	2 (4)	1 (4)	1 (5)
Other, n (%)	7 (14)	6 (23)	1 (4)
Education level			
Secondary education, n (%)	18 (35)	7 (27)	11 (44)
Tertiary/further education, n (%)	33 (65)	19 (73)	14 (56)
BMI (kg/m^2^), mean(SD)	30.4 (6.8)	31.3 (8.0)	29.4 (5.2)
Number of comorbidities (over and above index condition), mean (SD)	2.6 (1.7)	2.6 (1.9)	2.6 (1.4)
Length of asthma diagnosis (years), median (IQR)	18.5 (8.6–28.6)	17.0 (8.6–27.8)	20.3 (9.7–28.6)

BMI, body mass index; SIMD, Scottish Index of Multiple Deprivation.

### Primary outcomes

#### Recruitment and retention)

**Figure 1:**
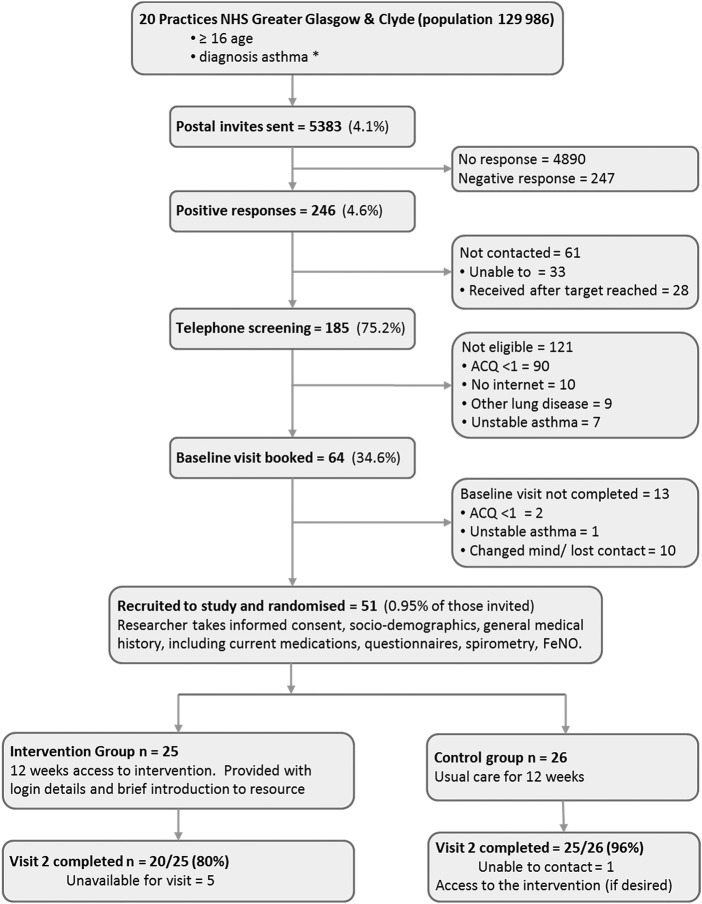
Flow of participants through study. *Actual search terms refined iteratively through recruitment (see online supplementary data file for detail). ACQ, Asthma Control Questionnaire score; FeNO, fractional exhaled nitric oxide; NHS, National Health Service.

Recruitment target of 50 participants was met (figure 1). Participating practices were mostly urban, and spread across deprivation categories. Response rate to the postal invitation was 4.6%, lower than anticipated, and only 27% of those screened were subsequently randomised, with the majority failing due to ACQ<1 (75%). Those randomised were younger (45.5 vs 51.5 years) and more likely to be female (75% vs 50%) than screen failures, but with similar socioeconomic deprivation. The attrition rate (not completing follow-up) was 12%: 20% in the intervention group, 4% in the comparison group (Fisher's test p=0.10).

#### Website use

Nineteen of the 25 participants in the intervention group logged in at least once (76%) with 17 going beyond the initial ‘core’ section. The subsequent section was tailored depending on which of three options was chosen: (1) I have never been prescribed a preventer inhaler (n=1); (2) I have been prescribed an inhaler but do not really use it (n=6); or (3) I have a preventer and usually use it as prescribed (n=10). The mean number of logins was 1.8 (range 0–7), median 1, (IQR 1–2), and the average time spent on the website during the study period was 18 min (range 0–48.9). More detail is shown in online supplementary figure A.

Beyond the core ‘introduction’ and ‘my asthma’ sections, the most popular sections were ‘take the 4-week challenge’ (n=13), and ‘common concerns and queries’ (n=11). Further usage data are shown in online supplementary table B. The majority (95%) of participants acknowledged that asthma was impacting on their life (online online supplementary table C).

#### ACQ score

Our planned analysis was for the seven-question version of the ACQ, which includes spirometry, for which there was considerable missing data (n=23; table 1). There was no significant difference in the intervention group compared with the control group (−0.42 (95% CI −0.95 to 0.11), p=0.121). We also analysed the equally valid six-question version (without spirometry)^24^ which was available for all (n=45), and demonstrated a similar result and it is this result which is presented in table 2.

**Table 2:** Primary outcomes (ACQ and mini-AQLQ)

		Intervention	Control	Estimated difference (95% CI)	p Value
ACQ score 6-question version (continuous 0–6; 0=totally controlled, 6=severely uncontrolled)					
Baseline	Mean (SD)	1.87 (0.59)	1.97 (0.68)		
Follow-up	Mean (SD)	1.22 (0.91)	1.65 (1.15)		
Change	Mean (SD)	−0.65 (1.08)	−0.32 (0.94)	−0.36 (−0.96 to 0.23)	0.225
ACQ score 6-question version (MCID improvement at follow-up)					
Improvement ≥0.5	n (%)	11 (55%)	12 (48%)		0.767
Mini-AQLQ score (continuous 1–7; 1=severely impaired; 7=not impaired at all)					
Baseline	Mean (SD)	4.97 (1.03)	4.65 (1.02)		
Follow-up	Mean (SD)	5.40 (1.01)	4.76 (1.30)		
Change	Mean (SD)	0.43 (0.78)	0.11 (0.88)	0.38 (−0.13 to 0.89)	0.136
Mini-AQLQ score (MCID improvement at follow-up)					
Improvement ≥0.5	n (%)	10 (50%)	9 (36%)		0.379

Summaries of scores at baseline, follow-up and change from baseline, with estimated between-group difference from baseline-adjusted linear regression model with 95% CI and p value. Summaries of achievement of an improvement by more than the MCID at follow-up, with Fisher's exact test p values to compare groups.

ACQ, Asthma Control Questionnaire (fall in score is desirable); AQLQ, Asthma Quality of Life Questionnaire (rise in score desirable); MCID, minimum clinically important difference.

Fifty-five per cent of the intervention group and 48% of the comparison group achieved the MCID of an improvement of at least 0.5 points (p=0.767).

#### AQLQ score

There was no significant difference in mini-AQLQ scores in the intervention group compared with the control group (table 2). Fifty per cent of the intervention group and 36% in the comparison group achieved the MCID of improvement of at least 0.5 points (p=0.379).

### Secondary outcomes

The rationale for including a range of secondary outcomes was to assess their feasibility for inclusion in any future full-scale RCT. All outcomes were acceptable to participants and feasible to measure and analyse, apart from spirometry.

#### Mini-AQLQ domain scores

The ‘activity limitation’ domain of the mini-AQLQ showed a statistically significant improvement in scores in favour of the intervention group (table 3). The remaining individual domains of the mini-AQLQ showed numerical improvement in the intervention group, which were not statistically significant.

**Table 3:** Secondary outcomes (continuous variables normally distributed)

		Intervention	Control	Estimated difference (95% CI)	p Value
Mini-AQLQ symptom domain score (continuous, 1=severely impaired; 7=not impaired at all)					
Baseline	Mean (SD)	4.56 (1.10)	4.30 (0.84)		
Follow-up	Mean (SD)	5.15 (1.20)	4.38 (1.35)		
Change	Mean (SD)	0.59 (1.10)	0.08 (1.05)	0.56 (−0.08 to 1.22)	0.084
Mini-AQLQ activity limitation domain score (continuous, 1=severely impaired; 7=not impaired at all)					
Baseline	Mean (SD)	5.30 (1.24)	5.31 (1.33)		
Follow-up	Mean (SD)	5.98 (0.92)	5.38 (1.33)		
Change	Mean (SD)	0.68 (1.01)	0.07 (1.10)	**0.60 (0.05 to 1.15)**	**0.034**
Mini-AQLQ emotional function domain score (continuous, 1=severely impaired; 7=not impaired at all)					
Baseline	Mean (SD)	5.48 (1.09)	4.80 (1.48)		
Follow-up	Mean (SD)	5.75 (1.01)	4.84 (1.82)		
Change	Mean (SD)	0.27 (0.78)	0.04 (1.30)	0.35 (−0.33 to 1.03)	0.301
mini-AQLQ environmental domain score (continuous, 1=severely impaired; 7=not impaired at all)					
Baseline	Mean (SD)	4.75 (1.39)	4.11 (1.54)		
Follow-up	Mean (SD)	4.85 (1.30)	4.23 (1.67)		
Change	Mean (SD)	0.10 (0.89)	0.12 (0.90)	0.08 (−0.46 to 0.62)	0.768
PAM (continuous, 0=no activation; 100=high activation)					
Baseline	Mean (SD)	65.7 (10.0)	66.2 (14.1)		
Follow-up	Mean (SD)	73.0 (13.9)	65.7 (16.5)		
Change	Mean (SD)	7.3 (11.3)	−0.5 (12.5)	**7.72 (0.53 to 14.90)**	**0.036**
MMAS (continuous, range 0–8, 0=low adherence; 8=high adherence)					
Baseline	Mean (SD)	4.88 (1.97)	5.59 (1.85)		
Follow-up	Mean (SD)	5.46 (1.80)	5.82 (1.85)		
Change	Mean (SD)	0.58 (1.37)	0.23 (1.03)	0.19 (−0.50 to 0.88)	0.586
MMAS (MCID improvement at follow-up)					
Improvement ≥2.0	n (%)	6 (30)	1 (4)		**0.034**
FEV_1_ (L) (continuous) (n=22)					
Baseline	Mean (SD)	2.62 (0.56)	2.66 (0.69)		
Follow-up	Mean (SD)	2.72 (0.58)	2.68 (0.49)		
Change	Mean (SD)	0.10 (0.18)	0.02 (0.31)	0.08 (−0.12 to 0.27)	0.428
FEV_1_% predicted (continuous) (n=22)					
Baseline	Mean (SD)	87.4 (13.6)	85.2 (17.1)		
Follow-up	Mean (SD)	90.6 (13.8)	85.7 (11.8)		
Change	Mean (SD)	3.3 (6.3)	0.6 (9.4)	3.4 (−2.8 to 9.5)	0.265
FEV_1_/FVC (%) (continuous) (n=22)					
Baseline	Mean (SD)	76.7 (7.0)	77.6 (10.9)		
Follow-up	Mean (SD)	79.1 (6.7)	80.2 (9.5)		
Change	Mean (SD)	2.4 (5.3)	2.6 (4.5)	−0.4 (−3.9 to 3.1)	0.829

Summaries of scores at baseline, follow-up and change from baseline, with estimated between-group difference from baseline-adjusted linear regression model with 95% CI and p value. Summaries of achievement of an improvement by more than the MCID at follow-up, with Fisher's exact test p values to compare groups. N=45 unless otherwise stated.

p Values in bold indicate significance <0.05.

ACQ, Asthma Control Questionnaire; AQLQ, Asthma Quality of Life Questionnaire; FEV_1_, forced expiratory volume in 1 s; FVC, forced vital capacity; MCID, minimum clinically important difference; MMAS, Morisky Medication Adherence Score; PAM, Patient Activation Measure.

#### Other patient-centred outcomes

There was a significant improvement in PAM scores (tables 3 and 4) in the intervention group compared with the control group, indicating that intervention patients were more highly activated in relation to managing their own health.

**Table 4:** Secondary outcomes (variables not normally distributed)

		Intervention	Comparison	p Value
EQ-5D health utility (continuous, 0.000=dead; 1.000 perfect health)				
Baseline	Median (IQR)	0.848 (0.725, 1.000)	0.796 (0.620, 1.000)	
Follow-up	Median (IQR)	1.000 (0.796, 1.000)	0.796 (0.727, 1.000)	
Change	Median (IQR)	0.000 (0.000, 0.111)	0.0000 (−0.052, 0.194)	0.972
EQ-5D visual analogue scale (continuous, 0 worst health; 100 best health)				
Baseline	Median (IQR)	75 (70, 84)	80 (70, 90)	
Follow-up	Median (IQR)	80 (73, 88)	80 (70, 90)	
Change	Median (IQR)	2.5 (−6.5, 13.0)	1.0 (−10, 10)	0.409
FeNO (continuous, low score indicates low inflammation)				
Baseline	Median (IQR)	26.0 (16.0, 46.5)	25.0 (11.0, 36.0)	
Follow-up	Median (IQR)	23.0 (12.0, 44.5)	19.0 (10.0, 27.0)	
Change	Median (IQR)	−2.5 (−11.5, 8.5)	−2.0 (−15.0, 2.0)	0.615
Puffs reliever taken per average week(continuous)				
Baseline	Median (IQR)	11 (7, 28)	4 (2, 12)	
Follow-up	Median (IQR)	5 (0.5, 14)	4 (0, 28)	
Change	Median (IQR)	−7 (−14, 1)	0 (−4, 4)	0.022
Percentage prescribed ICS reportedly taken (continuous)				
Baseline	Median (IQR)	85.7 (14.3, 100.0)	100.0 (71.4, 100.0)	
Follow-up	Median (IQR)	92.9 (71.4, 100.0)	100.0 (85.7, 100.0)	
Change	Median (IQR)	0.0 (0.0, 14.3)	0.0 (0.0, 7.1)	0.730
Equivalent beclometasone dose (μg) (continuous)				
Baseline	Median (IQR)	400 (300, 1000)	800 (400, 800)	
Follow-up	Median (IQR)	400 (300, 1000)	800 (400, 800)	
Change	Median (IQR)	0 (0, 0)	0 (0, 0)	0.209
Prednisolone course	n (%) with at least one	1 (5)	3 (12)	0.617
Hospital/A&E visit	n (%) with at least one	0	0	–
Non-routine GP/nurse visit	n (%) with at least one	3 (15)	6 (24)	0.710
Routine GP/nurse visit (eg, asthma review)	n (%) with at least one	5 (25)	8 (32)	0.745

Summaries of scores at baseline, follow-up and change from baseline. Summaries of prescribing and health service use over the study period, with Fisher's exact test for categorical data, and Mann-Whitney test to compare median differences between groups.

p Values in bold indicate significance <0.05.

A&E, accident and emergency; ACQ, Asthma Control Questionnaire; AQLQ, Asthma Quality of Life Questionnaire; EQ-5D, EuroQol; FeNO, fractional exhaled nitric oxide; GP, general practitioner; ICS, inhaled corticosteroids.

There was no significant difference in mean MMAS scores in the intervention group (table 3) compared with the control group. However, more participants in the intervention group achieved the MCID≥2 compared with usual care (30% vs 4%, p=0.034), although the intervention group did have lower baseline scores.

The change in EQ-5D health utility score showed no significant between-group difference (table 4), with median change in score of 0 in both groups.

#### Physiological and inflammatory outcomes

Spirometry analysis included only those meeting ATS acceptability standards (22/45, 11 per group).^22^ Effect sizes were small, and none achieved statistical significance (table 3).

FeNO levels (indicating airways eosinophilic inflammation) showed no significant between-group difference (table 4).

#### Medication changes and health service contacts

The median weekly number of puffs of reliever inhaler used in the intervention group reduced from 11 to 5, but remained unchanged in the control group at 4 puffs per week at baseline and at follow-up (p=0.022) (table 4). Although this between-group change in bronchodilator use was statistically significant, the groups were imbalanced at baseline. There was no significant between-group difference in the percentage of recommended ICS doses self-reportedly taken, nor the equivalent beclometasone doses prescribed. There were no significant between-group differences in health service contacts or prednisolone courses prescribed.

#### Further feasibility outcomes

The PETS results are shown in online supplementary table A, illustrating barriers to using the website. The biggest barriers relate to time and opportunity, rather than content.

No serious adverse events were recorded.

The main source of missing data was from the spirometry results where 23 participants had results not suitable for analysis, due to not meeting ATS criteria. All questionnaires were completed sufficiently well to allow calculation of scores, with only one response missing from each of the mini-AQLQ, PAM and MMAS all from different participants.

#### Sample size for a fully powered subsequent study

Using baseline-adjusted calculations of the change in ACQ score above assuming a SD of 1.0, a sample size of 134 would be required to detect a between-group change of ≥0.5 (MCID) in ACQ with 90% power at 0.05 significance. Assuming a similar attrition rate of 12%, the total sample size required would be 154.

## Discussion

### Principal findings

This phase 2 pilot RCT of the Living well with Asthma resource demonstrates that this website merits further development, and that subsequent progression to a full-scale phase 3 RCT is feasible. Recruitment targets were achieved, and attrition rates were comparable to rates of other published digital interventions.^9^ We had no upper age limit, unlike similar asthma digital intervention studies. This is important as our recent metareview only found one study that included participants over 50 years of age, and descriptions of participants’ characteristics were limited, with socioeconomic status ignored.^9^ This information is important to understand the ‘reach’ of the intervention.

In terms of primary efficacy outcomes, there were no significant between-group differences in terms of ACQ and mini-AQLQ, although it is important to note that this pilot trial was not powered to show such differences. However, there are some interesting findings in analysis, as both the ACQ and mini-AQLQ demonstrate encouraging and consistent trends in favour of the intervention group, with one subdomain of the AQLQ (activity limitation) reaching the MCID and statistical significance. It is worth noting that for both primary efficacy outcomes, a proportion of those in the comparison group demonstrated an improvement in MCID scores as well as the intervention group. This is often the case in unblinded complex intervention trials, and validates our approach of making this a pilot RCT, and not just a feasibility study. In terms of website use, 76% of individuals logging in is comparable with other behaviour change websites,^25^ ^26^ and it is encouraging that an average of only 18 min usage resulted in consistently positive trends across almost all outcomes. Asthma-specific research indicates that users like to spend 5–8 min per online session.^27^ Our exploration of usage patterns suggests that some users missed sections that they could potentially have benefited from. These two facts combined lead us to conclude that it would be preferable to provide the core modules initially and then ‘release’ further sections weekly or fortnightly, a strategy that has been used successfully for a weight loss intervention also developed using LifeGuide software.^26^ Qualitative process evaluation interviews of those in the intervention group have been completed and will be reported separately. Findings from this qualitative work will inform the further development of this resource, prior to evaluation in a full-scale trial.

We assessed the feasibility of collecting a range of secondary outcomes in any future RCT, and in doing so demonstrated a significant improvement in the PAM, which indicates that those in the intervention group had improved knowledge, confidence and skills to manage their asthma. Significant between-group differences in the numbers of patients showing a MCID improvement in adherence and reliever use should be interpreted with caution due to baseline between-group imbalances. The feasibility of researchers undertaking spirometry in the participants’ own homes using a portable handheld device was found to be low, as reported in other studies.^28^ Potential solutions include more intensive training of research staff; use of a device providing test-by-test acceptability information or undertaking trial visits in a dedicated clinical research facility by staff experienced in spirometry. However, this latter solution could have a negative effect on recruitment, as 21% of our study visits were undertaken in the evening and weekend, which facilitated recruitment of a population who can rarely make it into such RCTs (full-time employed). There is a balance between precision of measurements versus encouraging a more representative sample. Whether spirometry is required at all in a study aimed at people with mild-to-moderate asthma is not clear, and there is precedence in the literature for not including these outcome measures in similar primary care-based trials or for using simpler to perform lung function measures such as peak expiratory flow rate.^29^ ^30^

Lack of time and opportunity were the biggest barriers to using the website and providing the contents on a smartphone app or tablet would be worth investigating. During the introduction questions at the start of the website, 95% of users agreed to statements which showed that asthma was negatively impacting on their lives. However, at the end of the trial, 42% of users doubted the personal relevance of the website, anecdotally reporting that the website would be more useful for people with symptomatic asthma. To be in the trial in the first place, all users were symptomatic (as defined by ACQ score), so challenging this mismatch between users’ perceptions and the reality would be warranted in future versions of a mobile friendly digital intervention.

### Strengths and limitations

Blinding to group allocation during analysis was not possible due to the different numbers in each group being known by the researcher undertaking the analysis. As with many digital interventions, the ‘reach’ is a potential issue and our low response rate is a concern, even taking into account our very broad recruitment strategy. Similar trials have described similar recruitment difficulties.^31^ However, given how common asthma is, improvements in even a small proportion of patients could lead to significant benefit overall, particularly with an intervention such as that trialled here which is entirely internet based and once developed is very economical to make available to large numbers of people. Therefore, what seems like a low reach can still improve outcomes for a large number of people. We have described our population in detail, and our baseline characteristics demonstrate that patients were recruited from a range of socioeconomic backgrounds. Those excluded due to not having internet access were older than those who were excluded for other reasons (data not shown), but this is becoming less of an issue with year-on-year increases in the number of households with internet access (84% in 2014, UK).^32^

### Comparable studies in the literature

Our recently published metareview suggests digital interactive interventions to support asthma self-management show promise, but there is no clear picture about the ‘active ingredients’ of the interventions.^9^ In the development of this intervention, we have described its contents fully including an analysis of behaviour change techniques used,^13^ allowing more meaningful future comparisons. When focusing on interventions aimed at those with mild-to-moderate asthma, most have included considerable health professional input as well as self-monitoring work on the part of the participants, and have not shown clinical improvements.^33^ This evaluation of Living Well with Asthma adds to the literature on digital asthma self-management suggesting that an intervention not including regular user self-monitoring or costly health professional input may have positive results.

### Future research

We have shown that evaluating the Living Well with Asthma intervention was feasible and resulted in encouraging trends in clinical outcomes. Further qualitative work to understand usage patterns with intervention group participants have been completed and will inform a future version of the resource. To overcome the ‘practical barriers’ to using the intervention, future versions need to be mobile and tablet compatible, and will require further user testing. Following this development work on the resource, these findings suggest that a large-scale phase 3 RCT is merited, with some exploration of recruitment strategies and minor modification to outcome measurement methods. Low-intensity digital interventions that are easier to deliver at scale may be a more successful strategy, particularly in those with mild-to-moderate asthma.
