# Supplementary material for: Findings from a pilot Randomised trial of an Asthma Internet Self-management Intervention (RAISIN)
Source: BMJ Open. 2016 May 12;6(5):e009254. doi: 10.1136/bmjopen-2015-009254 (PMC4874112; doi:10.1136/bmjopen-2015-009254)
Supplement: Supplementary data [file bmjopen-2015-009254supp.pdf]

ONLINE SUPPLEMENTARY DATA FILE FOR THE RAISIN STUDY RCT

Contents:

1. Search terms used for identifying participants from primary care clinical databases (pg1)
2. Screenshot sample from 4 week challenge page (pg2)
3. Table A: Problematic Experiences of Therapy (PETS) Barriers to following website advice (pg3)
4. Table B: How often different sections were visited and for how long (pg4)
5. Table C: User responses regarding impact of asthma on life (pg4)
6. Figure A: Total time logged in and number of logins per user (pg5)

Search terms for identifying potential participants

The original strategy had 2 terms:

Asthma

AND ≥ 16 years

However, we were identifying many asymptomatic individuals and therefore we iteratively refined the search to focus on patients who were symptomatic in that they had requested a reliever inhaler in the last 8 months and were not on any COPD specific inhalers (at the time of the search these were not being used for asthma) and excluding those on palliative care registers or on regular oral prednisolone. By the final search this has been modified to:

Asthma (active problem)

AND ≥ 16 years

AND salbutamol or terbutaline inhaler (in preceding 8 months)

NOT Spiriva, tiotropium (COPD specific inhalers)

NOT oral prednisolone (on current repeat medication)

NOT palliative care register.

<u>Screen shot from 4 week Challenge section.</u>

Living well with asthma

My asthma and treatments | Asthma review and action plans | Common concerns and queries | Exercise, stress and anxiety | Take the 4 week challenge | Like to stop smoking? | Useful links and info

Step 2 - What are the barriers to taking your inhalers?

Step 1 | **Step 2** | Step 3 | Getting started

Why do you find it hard to take your preventer as prescribed?

Think about this now before you start means you are more likely to manage to keep taking your inhaler for the full 4 weeks.

Listed below are some examples of barriers to taking inhalers that others have told us about.

Tick any that apply to you - one in each box.
Add any others that you can think of.

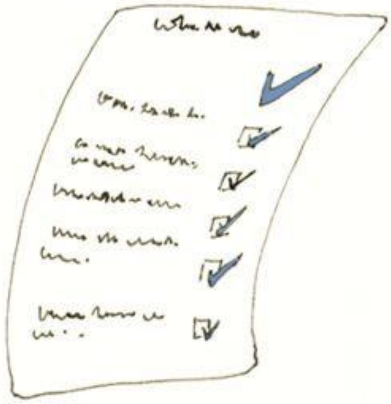

- ☐ I always forget my night time dose
- ☐ I am always so tired last thing I just want to get to bed
- ☐ I don't have time first thing in the morning, I am too rushed
- ☐ I just can't get into a routine with my inhaler
- ☐ Any other problems you might come up against? Type in the box below.

back | next

Problematic Experiences of Therapy (PETS)

Table A. Problematic Experiences of Therapy (PETS). Barriers to following website advice, range 1-5. Intervention group only (n = 19).

**Barrier:**	**Median (IQR)**
Symptoms too severe to follow website advice, or symptoms aggravated by website advice	1.0 (1.0 to 1.0)
Uncertain how to follow the website advice	1.0 (1.0 to 2.0)
Doubt about personal relevence of website advice	1.0 (1.0 to 1.7)
Practical obstacles to following website advice (e.g time, opportunity)	3.3 (2.0 to 4.0)

1 = strongly **disagree** with statement; 5 = strongly **agree** with statement

Website Use –Tables B & C and Figure A.

Table B. (Online Appendix) How often different sections were visited and for how long.

Topic	Total time spent (mins)	Number of visits to section*	Number of users visiting section
Introduction page (including home page) †	127.9	19	19
My Asthma (total)† ‡	76.8		
No preventer	12.5	2	2
Sometimes preventer	16.6	7	7
Usually preventer	47.7	16	10
Treatments	17.1	7	3
Asthma Reviews§	30.0	7	7
Action Plans	19.4	6	5
Physical Activity	46.0	13	9
Common concerns and queries	20.2	15	11
Stress & Anxiety	6.0	4	3
Take the 4 week Challenge	57.4	17	13
Like to stop smoking?ǁ	1.0	3	2
Useful info and links	22.7	11	9

* most number of visits to the introduction pages of a section
† Users were tunnelled through these sections at initial login
‡ Users had to chose one of three options to progress through this section
§ 6 users visited quiz within this section
ǁThis section only consisted of 1 page which linked to an external smoking cessation website, also developed using LifeGuide software [1, 2].

Table C. (Online Appendix) User responses regarding impact of asthma on life, n = 19. Users could choose more than one.

Questions asked by website:	Yes n(%)
1. Does your asthma ever stop you doing things you would like to do? (exercising, working, gardening, housework, visiting friends for example)	10 (53)
2. Does it sometimes affect your sleep?	12 (63)
3. Do coughs and colds sometimes cause your asthma to flare up?	16 (84)
4. Do you often have to use your blue/reliever inhaler more than twice a week?	15 (79)
5. Have you had an asthma attack (e.g. needing steroid tablets) in the last 6 months?	1 (5)
Users ticking at least one limitation due to asthma, n (%)	18 (95)

Figure A. (Online Appendix) Total time logged in and number of logins per user (in order of length of time on website).

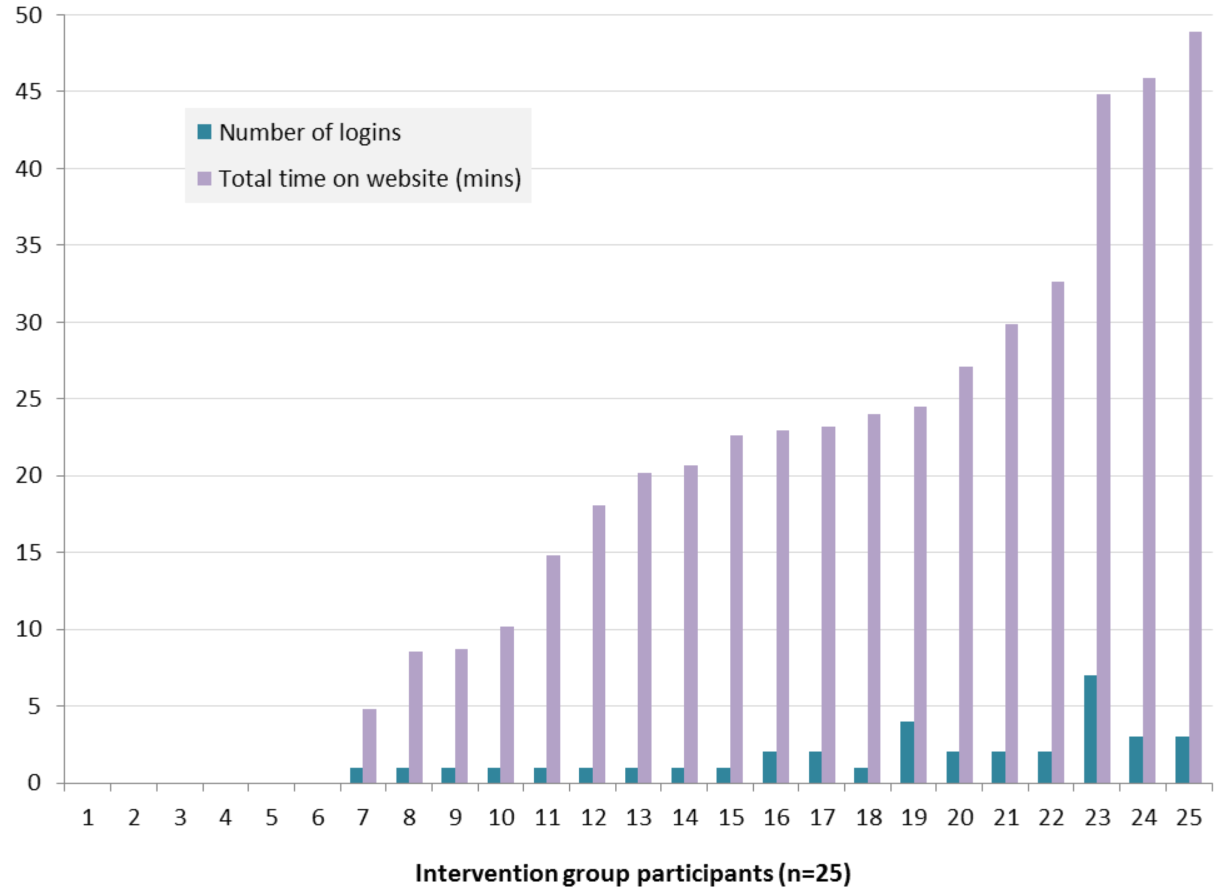

Supplementary File references

1. Michie S, Brown J, Geraghty AW, et al. Development of StopAdvisor: A theory-based interactive internet-based smoking cessation intervention. Transl. Behav. Med. 2012;**2**(3):263-75 DOI: 10.1007/s13142-012-0135-6
2. Brown J, Michie S, Geraghty AW, et al. Internet-based intervention for smoking cessation (StopAdvisor) in people with low and high socioeconomic status: a randomised controlled trial. Lancet Respir Med 2014;**2**(12):997-1006 10.1016/S2213-2600(14)70195-X
